# GePIF4 Increases the Multi-Flower/Capsule-Bearing Traits and Gastrodin Biosynthesis in *Gastrodia elata*

**DOI:** 10.3390/plants14111684

**Published:** 2025-05-31

**Authors:** Yue Xu, Zhiqing Wu, Yugang Gao, Pu Zang, Xinyu Yang, Yan Zhao, Qun Liu

**Affiliations:** 1 Laboratory of Medicinal Plant Cultivation and Breeding, National Administration of Traditional Chinese Medicine, College of Chinese Medicinal Materials, Jilin Agricultural University, Changchun 130118, China; xuyuejlau@163.com (Y.X.); 18434376791@163.com (Z.W.); zangpu@163.com (P.Z.); yangxinyu0413@163.com (X.Y.); lq1990@cnbg.net (Y.Z.); 2 Jiangsu Key Laboratory for the Research and Utilization of Plant Resources, Institute of Botany, Jiangsu Province and Chinese Academy of Sciences, Nanjing 210014, China

**Keywords:** *Gastrodia elata*, transcriptome analysis, *GePIF4*, high flower/capsule-bearing traits, gastrodin

## Abstract

The degeneration of germplasm is a key factor limiting the yield and quality of *Gastrodia elata* Blume. Sexual reproduction is a primary method to address this degeneration, while the number of flowers and capsules is directly related to sexual reproduction. However, the genetic mechanisms underlying the high flower/fruit-bearing traits in *G. elata* remain unclear. We first compared the quantitative and qualitative traits during the flowering to fruiting period of *G. elata*, including bolting height, flowering quantity, flowering time, fruiting quantity, capsule spacing, seed quality, etc. The natural materials were selected by multi-capsule and few-capsule for transcriptome analysis to screen the differentially expressed genes (DEGs); the candidate gene *GePIF4* was suspected to regulate the formation of multiple flowers and fruits. It was confirmed that GePIF4 has multiple biological functions in the overexpression of transgenic lines, including increasing numbers of vegetative propagation corms (VPCs) and promoting the growth of *G. elata*. Through comparative transcriptomic analysis of EV and OE-GePIF4 transgenic lines, the transcriptional regulatory network of GePIF4 was identified, and transient expression of GePIF4 was demonstrated to significantly promote gastrodin accumulation. The dual-LUC assay and in vitro yeast one hybrid results showed that GePIF4 could directly bind to GeRAX2 to regulate multi-capsule formation, and GePIF4 could directly bind to GeC4H1 to promote gastrodin accumulation. Therefore, we elucidate the role of GePIF4 in multi-capsule formation and secondary metabolite accumulation, thereby laying the groundwork for the genetic improvement of *G. elata* germplasm resources.

## 1. Introduction

*Gastrodia elata* Blume (Tianma), a member of the *Orchidaceae* family, is a traditional Chinese medicinal herb known for its ability to calm seizures, subdue liver yang, dispel wind, and unblock meridians, exhibiting various pharmacological effects [1,2]. The main functional active ingredients in *G. elata* are well known, including gastrodin and its related compounds (hydroxybenzyl alcohol, Parishin A, Parishin B, Parishin C, and Parishin E) [3]. They were mainly biosynthesized through the phenylalanine pathway under the action of phenylalanine ammonialyase (PAL), cinnamate 4-hydroxylase (C4H), trans-cinnamate 4-monooxygenase (CYP73A 4CL), shikimate O-hydroxycinnamoyltransferase (HCT), 5-O-(4-coumaroyl)-D-quinate 3′-monooxygenase (C3H), caffeoyl coenzyme A-O-methyltransferase (CCoAOMT), alcohol dehydrogenases (ADH), 3 β-glucosyltransferase (GT1), cis-zeatin oglucosyl-transferase (GT2), and hydroquinone glucosyltransferase (GT3) [4,5]. *G. elata* is a fully mycoheterotrophic plant, and its growth and development are primarily influenced by “two fungi and one type” (seed, *Mycena*, and *Armillaria*), which can reproduce both sexually and asexually [3]. In sexual reproduction, the life cycle encompasses several stages: from seed germination to the formation and differentiation of the primary corm, to the development of stem varieties like juvenile tubers, immature tubers, and mature tubers, culminating in bolting, flowering, and seed formation [6]. Most of these stages require the involvement of *Mycena* and *Armillaria*, with seed germination depending on nutrients provided by *Mycena*, while the other stages rely on *Armillaria* for nourishment, except for breeding the next generation, including bolting, flowering, and seed formation [3]. Asexual reproduction occurs through the differentiation of immature tubers into mature tubers. This method has a short production cycle, is easy to perform, has a rapid reproduction speed, and is cost-effective. However, after three generations of asexual reproduction, quality degradation may occur, leading to reduced yield and weakened disease resistance [6]. Therefore, sexual reproduction is the main way for *G. elata* to overcome the degradation of asexual reproduction, but relatively few reports exist on the sexual reproduction of *G. elata*.

Sexual reproduction begins with flowering regulation, which is mainly influenced by photoperiod/light signaling, vernalization/low temperature, hormone-signaling pathways, and other factors [7,8,9]. Light-signaling components such as GI (GIGANTEA) and ELF3 directly or indirectly affect the expression of downstream genes, promoting plant adaptation to the environment [10,11]. FT (Flowering LOCUS T) integrates light and temperature signals to regulate flowering response. For example, FT protein interacts with FD (Flowering LOCUS D) to form the FT-FD complex, activating the expression of SOC1 (SUPPRESSOR OF OVEREXPRESSION OF CONSTANS 1), AP1 (APETALA 1), and LFY (LEAFY) genes in the inflorescence meristem of plants, thereby promoting flowering [12,13]. Vernalization (low temperature) mainly regulates flowering by silencing the epigenetic mechanism of the FLC (Flowering LOCUS C) gene. FLC can inhibit the expression of downstream integrated genes such as FT and SOC1 [14]. In addition to the vernalization effect, changes in environmental temperature itself can also affect the flowering time of plants through the heat-sensing pathway. Suitable environmental temperature can induce the expression of the PIF4 (PHYTOCHOME INTERACTING FACTOR 4) gene in plants and inhibit the expression of the SVP (SHORT VEGETAIVE PHASE) and FLC genes, thereby promoting the expression of FT and SOC1 genes, and thus promoting plant flowering [15]. After sensing the gibberellin (GA) signal, GID1 (GA Insensitive DWARF 1) interacts with DELLA protein and promotes its ubiquitination degradation, thereby enhancing the expression of downstream SOC1 and FLY genes and promoting the plant flowering process [16]. In addition, plant hormones such as jasmonic acid (JA), brassinolide (BR), and abscisic acid (ABA) have been widely reported to be involved in flowering regulation. However, there are no reports of related flowering functional genes in *G. elata*, and the molecular mechanism of *G. elata* flowering regulation is in a blank state.

The regulation of flowering period branches contains three main pathways, including the RAMOSA pathway in maize (*Zea mays* L.), the ABERRANT PANICLE ORGANIZATION pathway in rice (*Oryza sativa* L.), and the COMPOSITUM pathway in barley (*Hordeum vulgare* L.), all of which have been shown to modulate species-specific inflorescence characteristics. The RAMOSA pathway in maize consists mainly of the RA1, RA2, and RA3 genes, while the APO pathway in rice is regulated by the interaction of F-box protein APO1 and the rice LEAFY homolog APO2. The COM pathway in barley includes two main regulatory factors, COMPOSITUM1 and COM2 [17]. In chickpea (*Cicer arietinum*), the SFL gene CaRAX1/2a, which encodes an R2R3-MYB transcription factor, specifically regulates the activity of secondary inflorescence meristems, leading to an increase in flower number [18]. In tomato (*Solanum lycopersicum*), the positive regulators of inflorescence branching, SISTER OF TM3 (STM3) and the negative regulator JOINTLESS 2 (J2), interact to regulate downstream target gene expression. STM3 activates downstream target genes, while J2 inhibits the transcription of these genes by forming a heterocomplex with STM3, preventing J2 from entering the cell nucleus. J2 also binds directly to the STM3 promoter to suppress its transcription, influencing STM3’s interaction with downstream genes and subsequently regulating the number of inflorescence branches in tomato [19]. Additionally, TARGET of EAT1 (SlTOE1) binds directly to the STM3 and tomato MADS-box gene 3 (TM3) promoters, inhibiting their expression and downregulating flower branching, while SlTOE1 can synergistically act with ENHANCER OF JOINTLESS 2 (EJ2) [20]. Therefore, these genes regulated by inflorescence branches can provide new ideas for the sexual reproduction breeding of *G. elata*.

In addition to flowering regulatory genes, the number of capsules also has great potential for improving sexual reproduction efficiency, such as the regulatory mechanisms of capsule length in sesame (*Sesamum indicum* L.), where a structural variation of 4.43 kb on chromosome 10 led to the deletion of the Siofp1 gene, which promoted an increase in capsule length. The trait of yielding multiple capsules per axil in sesame is controlled by a dominant gene [21]. The SiACS8 gene can increase the number of capsules on sesame stems, thereby enhancing yield [22,23]. Additionally, AtACS8 is involved in the biosynthesis of ethylene, indicating that the number of capsules on sesame stems is regulated by plant hormones in *Arabidopsis* [24]. SlMYB72 influences the degradation of the tapetum layer and pollen development in tomato by transcriptionally activating SlATG7 and autophagy, thus indirectly affecting seed quantity [25]. In *Arabidopsis*, PIN3 positively regulates the late initiation of ovule primordia, increasing the number of ovules and thereby promoting seed quantity to enhance reproductive performance [26]. The overexpression of Growth, Development and Splicing 1 (GDS1) promotes nitric oxide (NO) signaling, absorption, and assimilation by affecting the expression of multiple NO regulatory genes, including Nitrate Regulating Gene 2 (NRG2), ultimately increasing seed yield [27]. Therefore, it is necessary to systematically track the flowering regulation and capsule growth and development of *G. elata* and to conduct in-depth analysis of differentially expressed genes using transcriptomics and other techniques.

This article first systematically tracks the flowering regulation and capsule growth and development of *G. elata* and uses transcriptomics to conduct in-depth analysis of differential genes. Our research has found that GePIF4 can promote growth and development, which provides a theoretical basis for increasing flowering regulation and capsule growth followed by consequently enhancing the sexual reproductive capacity of *G. elata*.

## 2. Results

### 2.1. Evaluation of Flowering and Capsule Quality of G. elata

**Figure 1:**
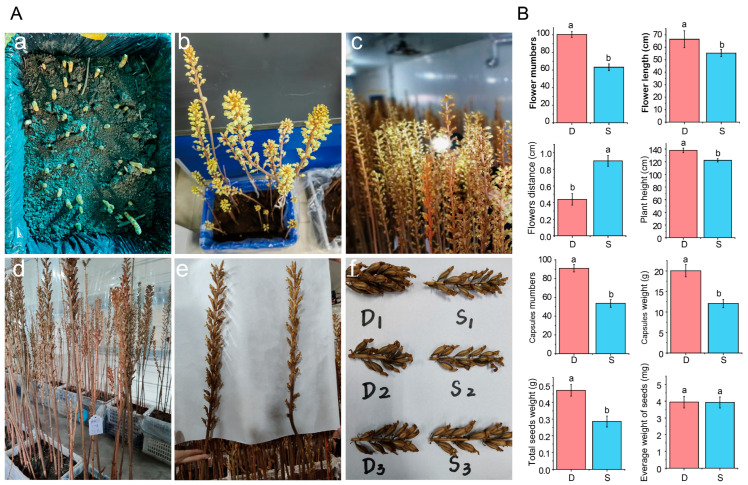
Evaluation of flowering and fruiting period of *G. elata*. (**A**) Growth status of the aboveground part of *G. elata* from April to May, including multi-flower and multi-capsule lines (D1–D3) and few-flower and few-capsule lines (S1–S3); (**a**), Mature tuber began to sprout; (**b**), Bolting and flowering; (**c**), Blooming stage; (**d**), Fruit ripening; (**e**), Comparison of multi-fruit and few-fruit; (**f**), Sample collection of multi-fruit and few-fruit *G. elata*. (**B**) Flowering quantity, flower distance, plant height, flower spacing, capsule quantity, capsule mass, total seed mass, and average seed mass of D and S lines. Different letters represent significant differences (*p* < 0.05).

Mature tubers (120 ± 5.0 g) were collected and planted indoors in March 2020. In early April, the mature tuber began to sprout (Figure 1(Aa)), and around April 15th, the flowering status was recorded (Figure 1(Ab)). At the end of April, the distance between flowers, plant height, and inflorescence length were recorded (Figure 1(Ac)). Male flowers from different plants were used for pollination for the capsules to gradually mature tubers (Figure 1(Ac,Ad)). Then, the number, weight, and total seed mass of capsules in each *G. elata* plant were recorded (Figure 1(Ae)). The results showed that *G. elata* exhibited trait segregation during the flowering and fruiting periods. The number of flowers, plant height, inflorescence length, number of capsules, capsule weight, and total seed weight were significantly higher in the multi-flower and multi-fruit lines (D) than in the few-flower and few-fruit lines (S). However, the flower spacing of the S lines was significantly higher than that of the D lines, and there was no significant difference in average seed weight, indicating that the seed number of the D lines was significantly higher than that of the S lines, but the difference between seeds of the D lines and S lines was not significant, suggesting that the sexual reproduction efficiency of the D lines was significantly higher than that of the S lines (Figure 1B). Therefore, in order to clarify the molecular mechanism of *G. elata* trait segregation, the D and S lines were selected for transcriptome analysis to screen candidate genes that regulate the segregation of multi-flower and multi-capsule formation traits in *G. elata*.

### 2.2. Transcriptome Sequencing, Functional Annotation, DEGs in GO, and KEGG Enrichment Analysis

From the transcriptome sequencing of AD_vs_AS, a total of 50.88 Gb of clean data was obtained from six samples, while the Q30 values exceeded 93.01% with an average GC content of 49.71%. The clean reads from the six samples were aligned with the assembled Transcript or Unigene library, resulting in mapped reads ranging from 20,422,249 to 27,167,133, and a mapped ratio of 81.59% to 85.74% (Supplementary Table S1). In total, 100,110 unigenes were obtained from the AD_vs_AS assembly, with an average length of 742.76 bp and a total length of 74,358,114 bp. The 100,110 transcriptome unigene sequences from the AD_vs_AS samples were annotated against eight databases, including NR, Swiss-Prot, and GO, resulting in a total of 23,996 unigenes with annotation information, which accounts for 23.97% of the total unigenes (Supplementary Table S2).

**Figure 2:**
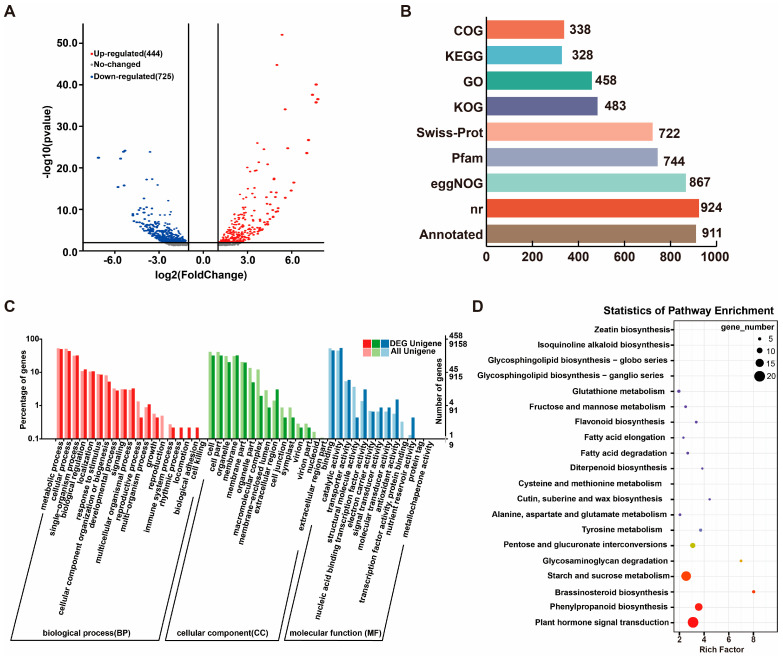
DEG analysis in AD_vs_AS. (**A**) Analysis of volcanic map of AD_vs_AS. (**B**) Annotations of DEGs in 6 databases. (**C**) DEGs in GO enrichment analysis. (**D**) DEGs in KEGG enrichment.

The differentially expressed genes (DEGs) in the AD_vs_AS comparison are shown in Figure 2A, including a total of 1169 DEGs, with 444 genes being upregulated and 725 genes downregulated (Figure 2A). In total, 931 DEGs were annotated to various databases, including NR, Swiss-Prot, GO, COG, KOG, KEGG, eggNOG, and Pfam. Among these, the highest number of annotations were found in the NR database, while the fewest were in the COG database (Figure 2B). The Venn diagram of four common databases illustrates the coverage of all annotated DEGs (Figure 2C, Supplementary Table S3).

Additionally, 168 DEGs were annotated to 91 KEGG metabolic pathways, primarily enriched in the following five categories: environmental information processing, metabolism, cellular processes, organismal systems, and genetic information processing. The KEGG enrichment analysis revealed that the top three KEGG pathways with the highest number of enriched DEGs were plant hormone signal transduction (11.9%), starch and sucrose metabolism (10.71%), and phenylpropanoid biosynthesis (8.33%) (Figure 2D). This indicates that the DEGs in the AD_vs_AS comparison are associated with multiple pathways, including plant hormone signaling, phenylpropanoid biosynthesis, and energy metabolism, reflecting a complex regulatory network (Figure 2D).

Based on the criteria of FC > 2.0 and FDR < 0.05, a total of 116 DEGs were identified as transcription factors (TFs) using transcription factor prediction tools. The transcription factor families include TFs, transcription regulatory factors (TRs), and protein kinases (PKs). These DEGs are members of TF families such as bHLH, AP2/ERF-ERF, NAC, TCP, and MYB. It is evident that the multi-fruit traits of *G. elata* are associated with multiple TFs. Combined with the results of TFs related to the multi-capsule traits of *G. elata*, it appears that bHLH, AP2/ERF-ERF, and NAC are particularly active in reproductive regulation (Figure S1).

### 2.3. Protein–Protein Interaction Network (PPI) Analysis and Screening for Related Core DEGs

**Figure 3:**
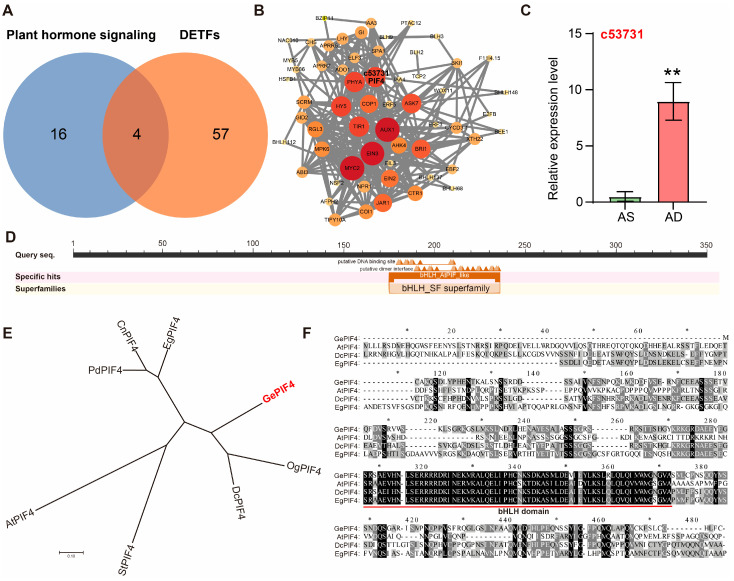
Screening and analysis of DEGs. (**A**) Venn analysis between plant hormone signal transduction DEGs (20) and DETFs (61). (**B**) PPI network analysis. (**C**) qRT-PCR analysis of c53731. (**D**) Structural domain analysis of c53731. (**E**) Phylogenetic tree construction of GePIF4 and homologous genes in different species: *G. elata* (GePIF4), *Arabidopsis thaliana* (AtPIF4), *Dendrobium catenatum* (DcPIF4), EgPIF4 (*Elaeis guineensis*), CmPIF4 (*Cinnamomum micranthum f. kanehirae*), CnPIF4 (*Cocos nucifera*), OgPIF4 (*Oncidium hybrid* cultivar), PdPIF4 (*Phoenix dactylifera*), and StPIF4 (*Senna tora*). (**F**) Amino acid sequence alignment among GePIF4, AtPIF4, DcPIF4, and EgPIF4, The bHLH DNA-binding domain is underlined in red. Values are expressed as means ± SE. Asterisks indicate significant differences as determined by Student’s *t*-test (**, *p* < 0.01).

According to the KEGG enrichment and transcription factor analysis results, Venn analysis was performed on the plant hormone-signaling pathway DEGs (20) and differentially expressed transcription factors (DETFs, 61). The results showed that there were four DETFs in the hormone pathway of DEGs: c25153.graph_c0 (ETHYLENE INSENSITIVE 3, EIL3), c53731.graph_c0 (PIF4), c54331.graph_c5 (auxin-responsive protein IAA4-like), and c43489.graph_c0 (Auxin-induced protein 22D) (Figure 3A). PPI analysis was conducted on these DEGs, and among the four common DEGs, c53731 (PIF4) was the key transcription factor connecting multiple functional genes (Figure 3B, Supplementary Table S4).

The qRT-PCR validation results were consistent with the transcriptome sequencing results, confirming the accuracy and reliability of the transcriptome data (Figure 3C). Based on the above results, the transcription regulatory network centered around the c53731 transcription factor may be a key transcription factor affecting the multi-flower and multi-capsule traits of *G. elata*. c53731 was successfully cloned and subjected to a bioinformatics analysis. c53731 belongs to the bHLH family and contains the conserved bHLH structural domain (Figure 3D). Phylogenetic tree construction and amino acid sequence alignment analysis showed that c53731 has the highest homology with PIF4 members in multiple species such as *Oncidium hybrid cultivar* and *Dendrobium catenatum*. Therefore, it was named GePIF4 (Figure 3E,F).

### 2.4. GePIF4 Actively Responds to Red Light Signals and Has Transcriptional Activation Function

**Figure 4:**
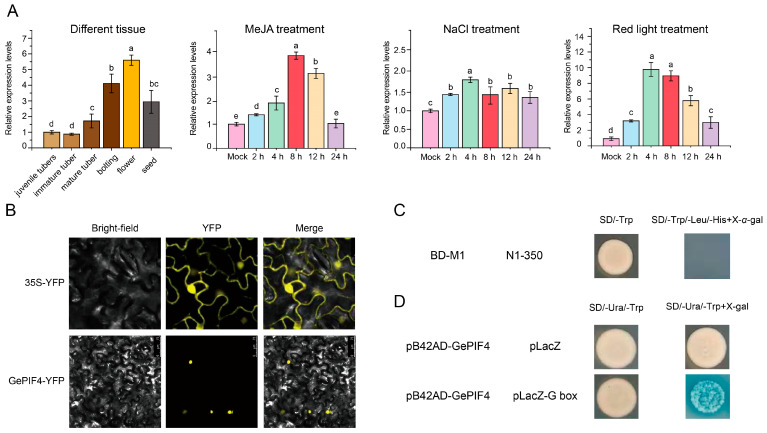
The nuclear localization transcription factor GePIF4 responds to different environmental signals and has transcriptional activation function. (**A**) Expression levels of GePIF4 in different tissues and under MeJA treatment, NaCl treatment, and red light treatment. (**B**) Subcellular localization analysis of GePIF4; the pHB-PIF4-YFP recombinant vector was used to transform *Agrobacterium* GV3101, and the *Agrobacterium* GV3101 containing pHB-YFP was used as a control. (**C**) Verification of transcriptional self-activation activity of GePIF4; pGBKT7-GePIF4 was transformed into Y2H yeast strains and grown in SD/-Trp select medium and then transformed to SD/-Trp/-Leu/-His+X-*α*-gal for detection of *β*-galactosidase activity. (**D**) Yeast one hybrid (Y1H) system was used to detect the transcriptional activation activity of GePIF4. The pB42AD-GePIF4 plasmid and pLacZ plasmid were co-transformed into EGY48 yeast, and pLacZ-G box was used to test whether GePIF4 could bind to G-box in vitro. Different letters indicate significant differences (*p* < 0.05) according to Duncan’s test.

In order to further analyze the function of GePIF4, the 2000 bp sequence of the *GePIF4* gene promoter was analyzed and cloned from the *G. elata* genome [28]. After online plantCARE analysis, the results showed that it contains multiple cis-acting elements, including light response (GA-motif, GATA motif, GC-motif, GT1-motif, GTGGC-motif, TCT motif), low-temperature responsiveness, gibberellin-responsive element (P-box), MeJA responsiveness (TGACG-motif), etc. (Figure S2). The qRT-PCR results showed that the expression level of *GePIF4* was highest during the peak flowering period, and red light induced a significant increase in *GePIF4* in response to MeJA hormone induction. The results indicate that GePIF4 actively participates in multiple signaling pathways (Figure 4A). Compared with MeJA and red light treatments, the *GePIF4* gene expression levels only reached 2-fold at 4 h after NaCl treatment and remained relatively stable (Figure 4A). We constructed a pHB-YFP plant expression vector containing the full-length *GePIF4* gene and transformed it into *Agrobacterium tumefaciens* GV3101, with subsequent injection into the back of tobacco leaves for 2–3 day and observation under confocal microscopy. Consistent with PIFs reported from other species such as AtPIF4, GePIF4 was only localized in the nucleus and belonged to the nuclear TF family compared with the control group (Figure 4B).

To further clarify whether GePIF4 has transcriptional activation function, GePIF4 segmented validation was conducted. The results showed that the full-length GePIF4 has no transcriptional self-activation function (Figure 4C). The yeast one hybrid system was used to detect the transcriptional activation activity of GePIF4. The pB42AD-GePIF4 plasmid and pLacZ plasmid were co-transformed into EGY48 yeast, and the pLacZ-G box was used as the experimental group to test whether GePIF4 could bind to the G-box in vitro. As shown in Figure 4D, GePIF4 could directly bind to the G-box motif in yeast, which indicated that GePIF4 can directly regulate the expression of downstream target genes positively/negatively by binding to the G-box on functional gene promoters. Based on the above results, we speculate that GePIF4 responds to various environmental signals and participates in downstream regulation. In order to clarify the biological function of GePIF4, we obtained transgenic lines of *G. elata* using the pollen tube pathway method and conducted subsequent evaluations.

### 2.5. GePIF4 Regulates the Formation of VPC Formation in G. elata

**Figure 5:**
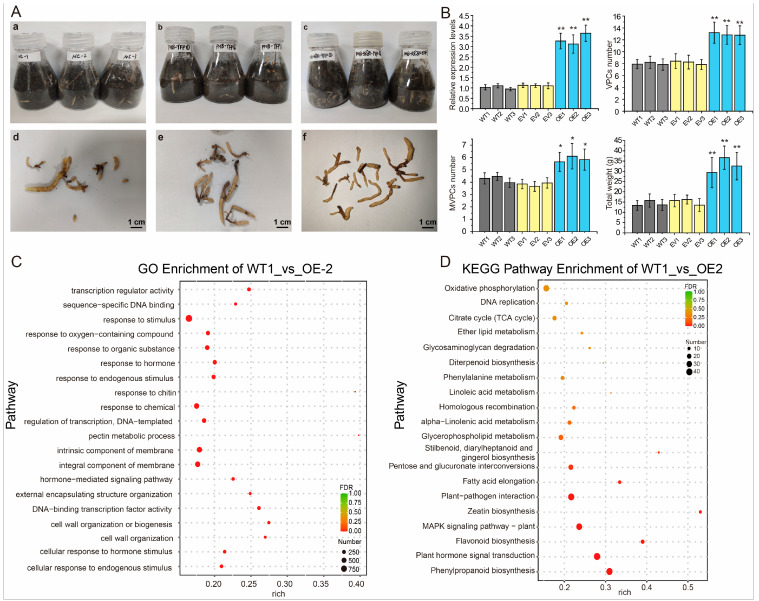
GePIF4 genetically modified *G. elata* material. (**A**) VPC obtained by pollen tube pathway method; (**a**,**d**) indicates the wild-type (WT) *G. elata*, (**b**,**e**) indicates the empty vector of pHB-YFP transgenic lines while (**c**,**f**) indicates the OE-GePIF4 transgenic lines. (**B**) Analysis of *GePIF4* expression level, VPCs, MVPCs, and total weight in WT lines and different transgenic lines. (**C**) GO enrichment of WT_1vs OE_2. (**D**) KEGG pathway enrichment of WT_1vs OE_2. “ns” indicates no significant difference (*p* > 0.05), * *p* < 0.05; ** *p* < 0.01. Yellow color dots indicate the FDR value between 0.25–0.75 while red color dots indicate the FDR value below 0.25. Different color indicates the 20 top significant enrichment pathways.

To verify the mechanism by which GePIF4 influences growth and development, the pollen tube pathway method was used for obtaining *GePIF4* overexpression lines (Figure 5A). The qRT-PCR results indicated that the expression level of *GePIF4* in the VPCs of the OE-*GePIF4* lines was significantly higher than that in the WT and OE-EV groups (*p* < 0.01). Further analysis was performed on the number of VPCs in the transgenic lines. The number of VPCs in the OE-*GePIF4* lines was significantly higher than that in the WT and EV groups (*p* < 0.01). Additionally, the number of MVPCs (multi-vegetative propagation corms) in the OE-*GePIF4* lines did not show a significant difference compared to the WT and EV groups (*p* > 0.05) (Figure 5B). Moreover, the total weight of the OE1-3 lines was significantly higher than that in the WT lines and EV lines. Therefore, the growth and development of GePIF4 transgenic *G. elata* was significantly higher than that of the WT and EV lines. To further clarify how GePIF4 regulates the growth and development of *G. elata*, we conducted transcriptomic analysis using WT1 and OE2 lines. In WT1_vs_OE_2, there are a total of 9030 DEGs, of which 4382 are upregulated and 4648 are downregulated (Figure S3). The GO results showed that GePIF4 was significantly involved in the stress response (Figure 5C). Meanwhile, the KEGG results indicate that GePIF4 mainly regulates polyphenol biosynthesis and hormone signaling (Figure 5D). In summary, GePIF4 participates in the growth and development of *G. elata* through various pathways.

### 2.6. Transient Expression of GePIF4 Significantly Promotes the Gastrodin Biosynthesis

**Figure 6:**
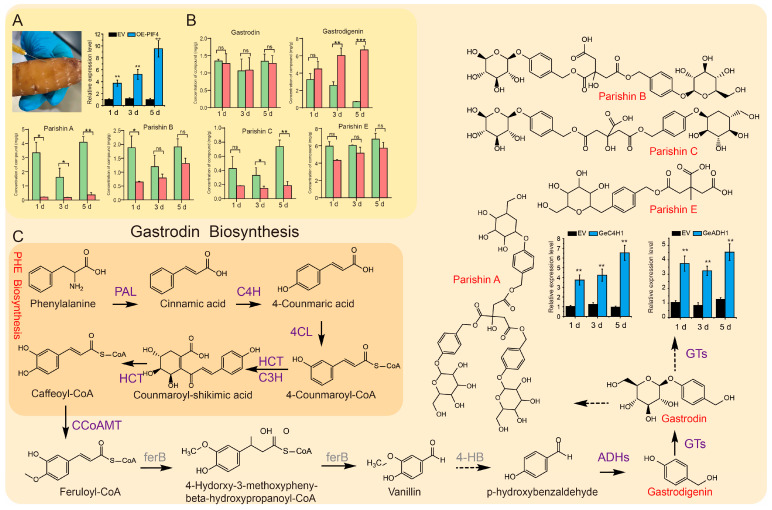
Establishment of transient expression system and determination of gastrodin content. (**A**) *G. elata* injection and PCR identification of GePIF4 gene expression level. (**B**) Content analysis of pharmacologically active components in *G. elata* at different transformation times following the transient expression of *GePIF4*: gastrodin; *p*-hydroxybenzyl alcohol; parishin E; parishin B; parishin C; parishin A; “ns” indicates no significant difference (*p* > 0.05); * *p* < 0.05; ** *p* < 0.01; *** *p* < 0.001. (**C**) Quantitative analysis of key enzyme genes and differentially expressed genes in the synthesis pathway of gastrodin.

To further verify the regulatory effect of GePIF4 on the metabolites of *G. elata*, a transient expression experiment was conducted in immature tuber, and the contents of gastrodin, *p*-hydroxybenzyl alcohol, Parishin A, Parishin B, Parishin C, and Parishin E were measured (Figure S4). The expression level of *GePIF4* in the tubers of the OE-*GePIF4* group on day 1 was significantly higher than that of the control group (*p* < 0.01). On day 3, the expression level of *GePIF4* remained significantly higher than that of the control group (*p* < 0.01) and was at a relatively high level. By day 5, the expression level of *GePIF4* continued to increase and remained at a high level, also significantly higher than that of the control group (*p* < 0.01) (Figure 6A). From day 1 to day 5, the contents of all six medicinal components in the transiently overexpressed immature tuber increased, with the content of *p*-hydroxybenzyl alcohol on days 3 and 5 being significantly higher than that of the control group (*p* < 0.01). Additionally, on day 5, the total content of gastrodin and *p*-hydroxybenzyl alcohol was significantly higher than that of the control group (*p* < 0.001) (Figure 6B). The overexpression of *GePIF4* positively influenced the total content of gastrodin and *p*-hydroxybenzyl alcohol in *G. elata*. Based on transcriptome data, seven gastrodin synthase DEGs were identified (Supplementary Table S5). qRT-PCR analysis was performed on *GeC4H1* and *GeADH1* genes, and the results showed that, from day 1 to day 5, the expression level of *GeC4H1* and *GeADH1* in the tubers of the OE-GePIF4 group was higher than that of the EV group. Moreover, the expression level of *GeC4H1* increased continuously for 1–5 days. Therefore, *GeC4H1* was a potential target for GePIF4 regulation of gastrodin biosynthesis.

### 2.7. GePIF4 Directly Binding to GeRAX2 and GeC4H1 Gene Promoter

**Figure 7:**
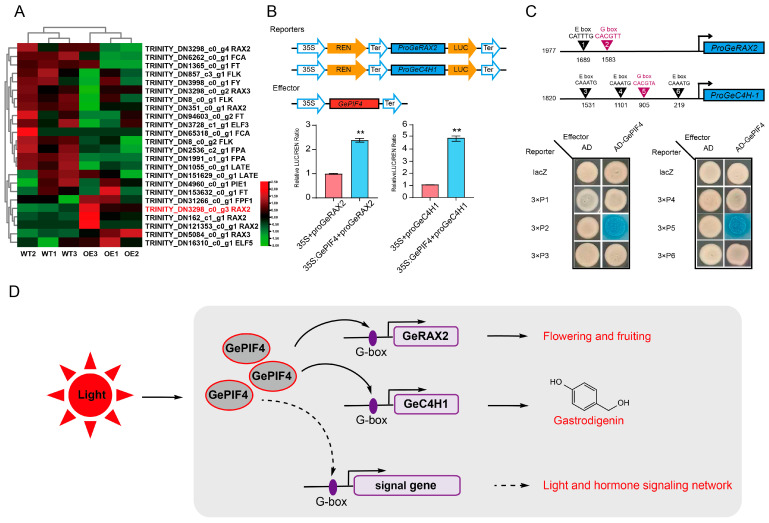
*GePIF4* directly regulates the expression of *GeRAX2* and *GeC4H1*. (**A**) Heatmap analysis of flowering-related genes. (**B**) Dual-LUC analysis indicated that GePIF4 increased the expression levels of *GeRAX2* and *GeC4H1*. (**C**) Y1H analysis indicated that GePIF4 directly binds to the G-box motifs in the promoters of *GeRAX2* and *GeC4H1*. (**D**) Model of GePIF4 regulates multi-flower/capsule-bearing traits and gastrodin biosynthesis in *G. elata*. Values are expressed as means ± SE. Asterisks indicate significant differences as determined by Student’s *t*-test (**, *p* < 0.01).

In order to further clarify how GePIF4 regulates the growth and development of *G. elata*, heatmap analysis was conducted on genes related to flowering and fruiting, and the expression of the *GeRAXs* gene was upregulated. Further analysis of cis-acting elements on *GeRAXs* and *GeC4H1* promoters revealed that both *proGeRAX2* (TRINITYDN3298_c0_g3) and *proGeC4H1* have an E-box and G-box (Figure S2). To investigate whether GePIF4 directly affects the expression levels of *GeRAX2* and *GeC4H1*, 35S-GePIF4 and pGreenII0800-proGeRAX2/proGeC4H1 recombinant vectors were constructed and subjected to dual-LUC assays in tobacco. The results indicated that GePIF4 increased the promoter activity of *GeRAX2* and *GeC4H1*, respectively (Figure 7B). The Y1H results further confirmed that GePIF4 activates the gene expression by binding to the G-box on the *GeRAX2* and *GeC4H1* promoters, thereby regulating the flowering and fruiting of *G. elata* and the biosynthesis of active ingredients.

Therefore, we derived a pattern diagram of GePIF4 regulating the growth, development, and accumulation of secondary metabolites in *G. elata* in response to multiple signals, especially for red light. GePIF4 promotes the formation of multi-capsule traits in *G.elata* by regulating TFs such as GeRAX2. On the other hand, GePIF4 promotes the accumulation of gastrodin by regulating phenylalanine pathways such as GeC4H1. In addition, GePIF4 may further participate in the growth and development of *G. elata* through the transcriptional regulation of hormone-signaling pathways (Figure 7D).

## 3. Discussion

### 3.1. The Physiological Data of G. elata from Flowering to Fruiting Lay the Foundation for Transcriptome Analysis to Reveal the Multi-Fruit Transcriptional Regulatory Network

The growth and development of *G. elata* is significantly different from that of photosynthesis plants [28]. It relies on the decomposition of lignin in decaying wood by *Mycena* and *Armillaria* underground to form polysaccharides and gastrodin, and its nutrient accumulation is independent from light regulation [3]. However, there are no relevant research reports on the sexual reproduction, flowering, and fruiting of *G. elata* on the ground. Therefore, this study first recorded the data of multiple flowers and multiple fruits/few flowers and few fruits in *G. elata* from bolting and flowering, capsule, and seed production, clarifying the relationship between multiple flowers and multiple fruits. Secondly, the traits of multiple flowers and multiple fruits/few flowers and few capsules were further screened, providing a basis for subsequent transcriptome determination.

The number of seeds is one of the important indicators of sexual reproduction in *G. elata* [6,29]. Therefore, we selected *G. elata* capsules containing mature seeds for separate sampling and transcriptomic analysis. We constructed a PPI network with 1116 DEGs and 61 DETFs and determined through heatmap analysis and qRT-PCR screening that the transcriptional regulatory network centered on PIF4 has a high correlation with *G. elata* seed quantity traits. Here, MYB72, PIN3, GDS1, NRG2, and other seed-development-related genes were not found, which may be due to species differences, screening specificity, and other factors [25,26,27]. In summary, we have provided candidate genes for subsequent functional gene validation by comparing transcriptome sequencing of differences in multi-capsule traits.

### 3.2. PIF4 Has Multiple Biological Functions and Has the Potential to Regulate Flowering, Fruiting, and Seed Quantity in G. elata

PIFs play an important role in plants’ response to biotic and abiotic stress [30]. In *Zea mays* L., ZmPIF3 transgenic plants showed increased relative water content, chlorophyll content, and chlorophyll fluorescence, as well as significantly enhanced cell membrane stability under stress conditions [31]. The expression of ZmPIF3 increased the tiller number and panicle number of transgenic rice without affecting grain yield [32]. ZmPIF3 plays an important role in ABA-mediated stomatal closure and the regulation of water loss, and it can improve drought resistance without affecting grain yield in transgenic rice [30]. Further research has shown that the heterologous expression of the maize PIF family gene ZmPIF3 enhances the drought resistance of transgenic rice [33]. In summary, ZmPIF3 is a promising candidate gene for water-saving and drought-resistant transgenic breeding and crop improvement in rice. Relatively speaking, there have been fewer reports on the functionality of PIF4. PIF4 regulates the levels of auxin and the expression of key auxin biosynthesis genes under high temperature, regulates plant stem elongation, and increases *Arabidopsis* resistance to high-temperature environments [34]. In cotton, the GhPIF4 gene is involved in regulating anther abortion under high-temperature conditions [35]. In this study, various experimental methods were used to evaluate the biological functions of GePIF4, such as amino acid comparison, phylogenetic tree construction, environmental/hormone treatment, subcellular localization, and transcriptional activation. Firstly, it was preliminarily determined that GePIF4 is consistent with PIF4 members in other species, with a bHLH structure and a phylogenetic relationship of over 75% (Figure 3E,F); significantly regulated by red light and MeJA hormones (Figure 4A), and serving as a nuclear localization transcription factor (Figure 4B); transcriptional activation studies have shown that GePIF4 does not have self-activating activity and can directly bind to the G-box and other functions in vitro (Figure 4C), laying the foundation for the subsequent acquisition and evaluation of transgenic *G. elata*.

### 3.3. GePIF4 Can Promote Gastrodin Accumulation and Multi-Flower/Capsule Formation in G. elata, Providing Target Genes for Breeding

C4H is a cytochrome P450-dependent monooxygenase that functions as a critical biosynthetic enzyme within the phenylpropanoid pathway [36]. The biosynthesis of downstream metabolites, including lignin, phenolic acids, and gastrodin, often depends on enzyme catalytic activity [37,38]. These secondary metabolites are essential for plant growth, development, and resilience against biotic and abiotic stresses [4]. KEGG enrichment analysis suggests that GePIF4 may enhance gastrodin production by modulating the expression of several enzyme-coding genes, including GeC4H1 (Figure 5D). Given the extremely low levels of gastrodin and its derivatives in nutritional propagation stems, which were below the detection threshold, transient expression assays were employed for evaluation (Figure 6A). These findings indicate that the transient expression of GePIF4 in *G. elata* significantly boosts the accumulation of key compounds, such as gastrodin (Figure 6B). The transcriptome analysis and qRT-PCR results identify GeC4H1 as a potential target of GePIF4 (Figure 6C). Further validation through dual-LUC and Y1H assays confirmed that GePIF4 directly enhances the expression of the *GeC4H1* gene, thereby facilitating the biosynthesis of gastrodin and p-hydroxybenzyl alcohol (Figure 7B,C).

By combining the transcriptome data of AD/AS with WT/OE, we collectively targeted several structural genes involved in flowering and fruiting, from which we identified the upregulated gene *GeRAX2* (Figure 7A). RAX2 is a RARA-MYB transcription factor that can increase the number of flowers [18]. Further validation through dual-LUC and Y1H assays confirmed that GePIF4 directly enhances the expression of the *GeRAX2* gene, thereby possibly promoting the formation of flower clusters in *G. elata* (Figure 7B,C). Therefore, we have preliminarily identified the biological functions of GePIF4 both in vivo and in vitro.

### 3.4. Functional Analysis of GePIF4 Transgenic Lines for Subsequent Research

PIF4 is a molecular hub in thermogenesis, which regulates thermogenesis by integrating light signals, circadian rhythms, and hormone signals, and has multiple biological functions [32,35]. For example, PIF4-ABI4 transcriptional activator complex synergistically enhanced seed dormancy by facilitating ABA biosynthesis and signaling [39,40]. In this study, the nuclear localization TF GePIF4 first responded to red light and various hormone signals and exhibited the transcriptional activation of downstream structural genes (Figure 4). Therefore, it occupies a relatively central position in the transcriptional regulatory network. PPI network analysis also indicates that GePIF4 is associated with auxin-induced protein 22D, TIFY 10a, GH3.8, ANT, bHLH18, PHL7, auxin transporter-like protein 2, TCP24, and IAA4 (Figure 3B). The good linear relationship between the co-expression of multiple transcription factors and auxin-signaling proteins suggests that GePIF4 may regulate the growth and development of *G. elata* at multiple levels. However, due to the long growth cycle of *G. elata* (3 years), we have not yet obtained data on genetically modified immature tubers or mature tubers, nor their subsequent flowering and fruiting [3,28]. Therefore, the mature tuber and flowering stage for OE-GePIF4 transgenic *G. elata* will be more beneficial for in-depth analysis in further study.

In conclusion, a total of 931 DEGs associated with the multi-capsule trait of the Changbai Mountain *G. elata* were identified. GePIF4 was successfully cloned and constructed in the recombinant overexpression vector pHB-*GePIF4*-YFP. Through the pollen tube pathway method, OE-GePIF4 transgenic *G. elata* were generated and characterized. GePIF4 was found to enhance the quantity of VPCs and promote branching by regulating cinnamacid-4-hydroxylase (C4H) to promote the biosynthesis of gastrodin, and to potentially regulate *VPCs* formation by modulating GeRAX2 and promoting the development of *MVPCs* properties. Our results preliminary elucidated the molecular mechanisms by which GePIF4 regulates the multi-capsule traits in *G. elata*, providing a theoretical basis for research on sexual reproduction in this species.

## 4. Materials and Methods

### 4.1. Plant Materials and Treatment

The immature tubers and mature tubers of *G. elata* were purchased from the planting base of Jingzhen *Gastrodia* Development Co., Ltd., located in Jingyu County, Jilin province, China (east longitude 126°30′–127°16′, north latitude 42°06′–42°48′). After flowering and pollination of the mature tubers, the number of flowers, plant height, and inflorescence length were recorded and analyzed. When the capsules formed, the number of capsules, capsule weight, and total seed weight were measured. The top capsules from plants with multi-capsule (AD) and few-capsule (AS) traits were quickly frozen in liquid nitrogen and stored at −80 °C, with three biological replicates set for each sample for transcriptome sequencing and qRT-PCR analysis. The immature tubers were used for transient expression studies. For subcellular localization and dual-LUC assays, *Nicotiana benthamiana* plants were cultivated in a greenhouse at 20 ± 3 °C under a stable photoperiod of 16:8 h (light/dark). Juvenile tubers, immature tubers, mature tubers, boltings, flowers, and seeds were collected for qRT-PCR analysis.

For red light treatment, we exposes the immature tubers to red light in a three-color incubator for 24 h; for NaCl and plant hormone treatment, 250 mM NaCl and 0.1 mM MeJA were injected into immature tubers. Samples were collected at 0, 2, 4, 8, 12, and 24 h, and the samples were analyzed using qRT-PCR.

### 4.2. RNA Sequencing Assembly and Analysis

The apex capsules of multiple-capsule (AD1, AD2, AD3) and few-capsule (AS1, AS2, AS3) *G. elata* and the WT1/2/3 and OE1/2/3 lines of the VPCs were obtained from frozen stocks. Approximately 100 mg of each sample was ground in liquid nitrogen, and total RNA was extracted using the RNAprep Pure Plant Total RNA Extraction Kit (Tiangen Biotech (Beijing) Co., Ltd., Beijing, China), following the manufacturer’s instructions. The purity of the RNA samples was assessed using a NanoDrop 2000 spectrophotometer (Thermo Fisher Scientific, Waltham, MA, USA), while the integrity and concentration of the RNA samples were accurately analyzed using an Agilent 2100 bioanalyzer (Agilent Technologies Santa, Clara, CA, USA). Qualifying total RNA was then utilized for transcriptome sequencing. For the qualifying samples, library construction was performed. Once the library was completed, preliminary quantification and insert size detection were conducted using a Qubit 2.00 Fluorometer and an Agilent 2100 bioanalyzer, respectively. After confirming library quality, transcriptome sequencing was carried out on the Illumina HiSeq high-throughput sequencing platform. Raw data were generated from the Illumina sequencing, which were subsequently filtered and processed to obtain clean sequences. The clean reads from the transcriptome were assembled using Trinity (v2.15.1) software accessed on 6 May 2022. Overlapping information among sequences was used to assemble overlapping groups (contigs), resulting in transcript sequences of varying fragment sizes and a simplified unigene set. The final unigene sequences were subjected to similarity comparisons using the Blast software (https://blast.ncbi.nlm.nih.gov/Blast.cgi (accessed on 6 May 2022); v2.14.0) against databases such as NR, Swiss-Prot, COG, KEGG, KOG, GO, Pfam, and eggNOG, thereby obtaining functional annotation information for all unigenes. Gene abundance matching and differential expression analysis of DEGs (differentially expressed genes) between multiple-fruit and few-fruit *G. elata* capsules were performed using FPKM (fragments per kilobase of transcript per million fragments mapped) values and DESeq2 (version 4.2) software. The Benjamini–Hochberg correction method was employed to adjust the significance *p*-values obtained from the hypothesis tests. DEGs were filtered based on the criteria of FC > 2 and FDR (false discovery rate) < 0.05, and the selected DEGs were subjected to enrichment analysis using the GO and KEGG databases. GO terms and KEGG pathways that met the criteria of FDR < 0.05 were defined as significantly enriched terms and pathways. The volcano map and heatmap were both completed by TBtools (v 2.210) [41].

### 4.3. qRT-PCR Analysis

*G. elata* RNA was extracted using plant RNA extraction kit (TARAXA, Sangon, Shanghai, China), followed by reverse transcription to obtain cDNA. Using the cDNA as a template, qRT-PCR validation was performed with a StepOne fluorescent quantitative PCR instrument and 2× SG Fast qPCR Master Mix. The real-time quantitative PCR primers for DEGs were designed using Primer 5.0 software, with the 18S gene serving as the internal control (Supplementary Table S6). The primers were synthesized by Sangon Biotech (Shanghai) Co., Ltd., Shanghai, China (https://www.sangon.com/ (accessed on 6 May 2022)). qRT-PCR detection was conducted using a two-step method (95 °C for 3 min; 45 cycles of [95 °C for 5 s, annealing/extending at 60 °C for 30 s]). Each reaction was performed in triplicate, and the relative expression levels of the differentially expressed genes were calculated using the 2^−ΔΔCt^ method [42]. qRT-PCR data were analyzed by Graphpad Prism 9.

### 4.4. Subcellular Localization

The full-length coding sequences of *GePIF4* were cloned into the pHB-GFP vector and sequenced by Sangon Biological Co., Ltd., Shanghai, China. The recombinant vectors pHB-GePIF4-GFP were transformed into *Agrobacterium* GV3101 using the freeze–thaw method, respectively. *Agrobacterium* containing the recombinant vectors were confirmed by PCR using primers 35S and GePIF4-R (Supplementary Table S6). *Agrobacterium* GV3101 containing pHB-GePIF4-GFP and the empty vector were initially cultured overnight in a 2 mL sterile centrifuge tube and subsequently transferred to a 50 mL sterile centrifuge tube until the optical density (OD_600_) reached 0.8–1.0. The well-cultivated *Agrobacterium* suspension was re-centrifuged and resuspended in a resuspension medium consisting of 10 mM MgCl_2_, 10 mM MES, and 100 μM acetosyringone. After incubation in the dark for 2–3 h, the backs of well-grown, 2-month-old tobacco leaves were injected with *Agrobacterium* and incubated in the dark for 48–72 h (Liu et al., 2025 [36]). The leaves were then observed using a confocal microscope (Zeiss LSM 900, Oberkochen, Germany).

### 4.5. Self-Activation Verification of GePIF4

The full-length coding sequences of *GePIF4* were individually cloned into the vectors pGBKT7 (*Eco*R I). Recombinant plasmids (pGBKT7) were transformed into yeast using the freeze–thaw method. Preliminary screening for positive strains was conducted on SD/-Trp solid medium. The positive strains were subsequently re-screened on SD/-Trp/-Leu/-His medium, and the re-screened strains were transferred to SD/Trp/Leu/-His+x-*α*-gal for blue–white screening based on *β*-galactosidase activity [36]. Six individual yeast colonies were analyzed as replicates.

### 4.6. Generation of Transgenic G. elata

Design primers were used to clone the full-length ORF of GePIF4 (Supplementary Table S6) and construct recombinant plasmids pHB-GePIF4-YFP with the overexpression vector pHB-YFP. These recombinant plasmids and empty vectors were transformed into *Agrobacterium* GV3101, and genetic transformation was carried out using the pollen tube pathway method to obtain transgenic *G. elata* lines. Using a syringe, *Agrobacterium* GV3101 containing the recombinant vectors and empty vectors (OD_600_ = 0.6–0.8) was injected into the pollinated floral columns, which were properly labeled. After 20 d, mature seeds were harvested and transferred to an incubator at 25 °C for co-cultivation with germinated fungi until the formation of vegetative propagation tubers, with cultivation conditions following the seed hydration method. Positive PCR identification was performed using the DNA from the obtained transgenic vegetative tubers as a template. For the OE-GePIF4 vegetative tubers, the primers PHB-GePIF4-F and YFPR were used (Supplementary Table S6). The PCR program was as follows: pre-denaturation at 94 °C for 1 min, denaturation at 94 °C for 30 s, annealing at 55 °C for 30 s, and elongation at 72 °C for 1 min per kb, for a total of 35 cycles.

### 4.7. Transient Expression of pHB-PIF4-YFP Agrobacterium in Immature Tuber

Recombinant plasmids pHB-GePIF4-YFP and pHB-YFP were injected into immature tubers using the *Agrobacterium* GV3101. The specific procedure is as follows: 5 mL of *Agrobacterium* suspension was injected into various sites on tuber, including the ring pattern, the basal umbilicus, and the apical growth cone. The injection depth was approximately 0.5 cm. The treated tubers were placed in a sterile foam box for dark incubation. Protein expression was observed using confocal microscopy at 1, 3, and 5 d after injection. The injected tubers were subsequently used for qRT-PCR and chemical composition analysis.

### 4.8. HPLC Determination of Gastrodin and Its Derivatives in G. elata

As for gastrodin, p-hydroxybenzyl alcohol, Parishin A, Parishin B, Parishin C, and Parishin E content analysis in *G. elata*, LC-10 AT high-performance liquid chromatography was used. The chromatographic column was Century SIL C18 (250 mm × 4.6 mm, 5 μm). The gastrodin, p-hydroxybenzyl alcohol, Parishin A, Parishin B, Parishin C, and Parishin E standards were used for calibration curves and correlation coefficients construction. Each transiently expressed sample (injected immature tubers were sliced, placed in an evaporating dish, boiled in water, steamed for 30 min, and dried at 60 °C; 2.0 g of each sample of *G. elata* powder was placed in a 50 mL conical flask with a stopper and added to 25 mL of dilute ethanol) was extracted using ethanol ultrasound (120 W, 40 kHz) for 30 min, then centrifuged and filtered with 0.22 μm microporous membrane to produce a sample with a loading amount of 20 μL. Column temperature was 30 °C; detection wavelength was 220 nm. Gradient elution conditions: mobile phase was acetonitrile (A), −0.1% phosphoric acid water (B). Gradient elution procedure: 0–10 min, 3–10% A; 10–15 min, 10–12% A; 15–25 min, 12–18% A; 25–40 min, 18% A [3].

### 4.9. Dual-LUC Assays

In the dual-luciferase assays, the pHB-GePIF4-YFP vector was constructed and subsequently introduced into *Agrobacterium* GV3101 to function as an effector, while pHB-GFP served as a negative control. The promoters of *GeC4H1* and *GeRAX2* were cloned and fused with the pGreen II 0800 vector, resulting in the pGreen0800-promoter recombinant vectors. These vectors were co-transformed into GV3101 to act as reporters. The effector and reporter strains were combined in a 2 mL proportion, incubated for 2 h, and subsequently injected into the leaves of 8-week-old *N. benthamiana* plants. After 48–72 h of cultivation in the dark, dual-LUC assays were conducted as previously described [36]. The LUC/REN (Firefly luciferase/Renilla luciferase) ratio was measured to assess expression levels, with three replicates performed for each experimental group.

### 4.10. Y1H Assays

Cis elements on the promoters of *GeC4H1* and *GeRAX2* were analyzed, focusing on the design of pLacZ vector primers targeted at the E-box and G-box sequences. After primer annealing, these were constructed into the pLacZ vector (*Eco*R I and *Xho* I). Additionally, the CDS sequence of PIF4 was constructed into the pb42AD vector (*Eco*R I). The recombinant pLacZ and pB42AD vectors were co-transformed into EGY48 gold yeast. Positive transformants were selected on SD/–Ura/–Trp media for 3 d at 30 °C and then transferred to SD/–Ura/–Trp+X-gal for blue and white spot screening [36].

## Data Availability

The authors confirm that the data supporting the findings of this study are available within the article.

## Supplementary Materials

The following supporting information can be downloaded at: https://www.mdpi.com/article/10.3390/plants14111684/s1, Figure S1. 61 differentially expressed transcription factors identified in multi-capsule transcriptome data. Figure S2. The analysis of gene promoters involved in this article. Figure S3. Volcanic map of differentially expressed genes in the nutritional propagation stem of transgenic *G. elata* overexpressing GePIF4. Figure S4. HPLC analysis of liquid phase diagram for the content of gastrodin and its derivatives in *G. elata* treated with transient expression of GeIPF4 and empty vector control groups. Supplementary Table S1. Sequencing overview and reads mapping. Supplementary Table S2. Unigenes functional annotation results statistics AD_vs_AS of *G. elata* capsule. Supplementary Table S3. GO enrichment analysis. Supplementary Table S4. Information of genes that relate to the formation of multi-capsule traits of *G. elata*. Supplementary Table S5. The seven Gastrodin synthase DEGs. Supplementary Table S6. qRT-PCR primer pair sequences for multi-capsule candidate genes.

## Abbreviations

ABA: abscisic acid; AD: multi-capsule; ADH: alcohol dehydrogenase; AP1: APETALA 1; AS: few-capsule; BR: brassinolide; C4H: cinnamate 4-hydroxylase; COM: COMPOSITUM; DEG: differentially expressed gene; EJ2: ENHANCER OF JOINTLESS 2; FD: flowering LOCUS D; FLC: flowering LOCUS C; FT: flowering LOCUS T; GA: gibberellin; GDS1: Growth, Development and Splicing 1; GI: GIGANTEA; GID1: GA Insensitive DWARF 1; J2: JOINTLESS 2; JA: jasmonic acid; LFY: LEAFY; NRG2: Nitrate Regulating Gene 2; PIF4: PHYTOCHOME INTERACTING FACTOR 4; PPI: protein–protein interaction networks; RAX: transcription factor RAX2; SlTOE1: TARGET of EAT1; STM3: SISTER OF TM3; SOC1: SUPPRESSOR OF OVEREXPRESSION OF CONSTANS 1; SVP: SHORT VEGETAIVE PHASE.
